# With thanks to our 2025 peer reviewers

**DOI:** 10.24095/hpcdp.46.3.06

**Published:** 2026-03

We are grateful to the following individuals for their significant contribution toHealth Promotion and Chronic Disease Prevention in Canadaas peer reviewers in 2025. Their expertise ensures the quality of our journal and promotes the sharing of new knowledge among peers in Canada and internationally.

Sarah Abdunnabi

Hanan Abramovici

Jeanne Alongi

Lynda G. Balneaves

Caroline Bergeron

Jacqueline Burt

Kiffer Card

Matthew Carroll

Stephanie Cerutti

Michelle Costa-Fagbemi

Alexis Crabtree

Jing Cui

Amanda Doggett

Gabriel John Dusing

Tonino Esposito

Benedict Fischer

Sally Fowler-Davis

Pamela Fuselli 

Samantha Goodman

Zoe Greenwald

John Gunn

Nicole Hammond

Franois-Olivier Hbert

Katya Herman

Melissa Holt

Joy M. Hutchinson

Michelle Kegler

Julia Kontak

Anita Koushik

Stephanie Lake

Scott Leatherdale

Julian Little

Elaina MacIntyre

Alyson Mahar

Kerry Mansell

Andrew McCabe

Ian McDowell

Steven McFaull

Lara McKenzie

Shweta Mital

Howard Morrison

Candace Nykiforuk

Rajna Ogrin

Udoka Okpalauwaekwe

Jennifer O'Loughlin

Heather Orpana

Heather Palis

Jasmine Pawa

Chelsea Pelletier

Sieara Plebon-Huff

Stephanie Prince Ware

Rose Ricciardelli

Katherine Rittenbach

Sarah Rondeaux

Amberley Ruetz

Adam Sherk

Sandra Slater

Danielle A. Southern

John Spence

Matthew Stackhouse

Valerie Tarasuk

Valerie Testa

Rania Tfaily

Samuel Tobias

Zachary Townsend

Mark Tremblay

Joslyn Trowbridge

Leigh Vanderloo

Mlanie Varin

Jennifer Vincent

Juliet Wakefield

Cliff Whetung

Rebecca F. Wilson

Meghan Winters

Scott Yamamoto

Chunhe Yao

